# Urinary strem-1 is an early outcome predictor for sepsis and sepsis-induced acute kidney injury

**DOI:** 10.1186/2197-425X-3-S1-A255

**Published:** 2015-10-01

**Authors:** HM Sherif, A Farghal, A Al Sisi, S Al Maraghy

**Affiliations:** Faculty of Medicine, Critical Care Medicine, Cairo University, Cairo, Egypt; Faculty of Medicine, Critical Care Medicine, Beni Suef University, Beni Suef, Egypt

## Background

Different biomarkers have been studied as outcome predictors in patients with sepsis, recent reports had indicated the usefulness of urinary soluble triggering receptor expressed on myeloid cells-1 (sTREM-1) as a better prognostic marker for sepsis and sepsis-induced acute kidney injury (AKI).

## Objectives

Detection of the prognostic value of urinary sTREM-1 in sepsis regarding the clinical course, development of AKI and the final outcome.

## Methods

Thirty critically ill patients (pts.) with sepsis (57.6 ± 7.5 y, 18 males) and a subset of 10 controls (45.6 ± 3.5 y, 7 males) were included in this study. Urinary sTREM-1 and C-reactive protein (CRP) serum levels were measured on admission, day 3 and 7, respectively. SOFA score was estimated at baseline and daily until discharge, death or up to 28 days. ICU length of stay, need for mechanical ventilation, vasopressors or renal replacement therapy, and development of AKI and the final outcome were recorded.

## Results

Compared to the controls, sTREM-1 in the patient group showed significantly higher values (3.78 ± 1.21 vs. 0.78 ± 0.16 ng/ml, *P* < 0.001). The patients who needed vasopressors (23 pts) or hemodialysis (pts 4) showed significantly higher sTREM-1 values than the other patients (4.06 ± 1.22 vs. 2.86 ± 0.51 ng/ml, *P* < 0.001 and 5.27 ± 1.2 vs. 3.55 ± 1.05 ng/ml, *P* < 0.05), respectively. Fair correlation could be detected between sTREM-1 and SOFA score on day 1 and 7 (r = 0.45, *P* < 0.05 and r = 0.47, *P* < 0.05), respectively, and with CRP values only on day 7 (*r* = 0.48, *P* < 0.05). Fair correlation could be detected between sTREM-1 and CRP only on day 7 (*r* = 0.46, *P* < 0.05). The ICU length of stay showed no correlation with sTREM-1 values. In patients who developed AKI (12 pts), sTREM-1 showed significantly higher values than those who didn't develop AKI (4.37 ± 1.34, 3.39 ± 0.95 ng/ml*, P* < 0.05). Compared to the survivors, the non survivors (14 pts) had significant higher sTREM-1 values (4.6 ± 1.14 vs. 2.96 ± 0.52 ng/ml, *P* < 0.001). The area under the curve (AUC) for sTREM-1 to predict AKI on day 1 was 0.73 (95% CI; 0.53-0.92), with best cutoff value of 4.02 ng/ml (sensitivity 66.7% & specificity 83.3%). The AUC for sTREM-1 to predict the ICU mortality on day 1 was 0.91 (95% CI; 0.81-1.01), with best cutoff value of 4.02 ng/ml (sensitivity 73.3% & specificity 100%).

## Conclusions

Urinary sTREM-1 could predict the clinical outcome, development of AKI and ICU mortality.

